# Effect of Cilostazol and Aspirin During Hyperacute Stroke Phase in Rats: An Experimental Research Study

**DOI:** 10.3390/neurolint17050069

**Published:** 2025-04-28

**Authors:** Christiana Anastasiadou, Anastasios Papapetrou, George Galyfos, Kostas Vekrellis, Patroklos Katafygiotis, Andreas Lazaris, George Geroulakos, Angelos Megalopoulos, Christos Liapis, Nikolaos Kostomitsopoulos, John Kakisis

**Affiliations:** 1 Department of Vascular Surgery, “George Papanikolaou” General Hospital of Thessaloniki, 570 10 Exochi, Greece; megalangelo@yahoo.com; 2 Department of Vascular Surgery, “KAT” General Hospital of Athens, 145 61 Kifisia, Greece; tas1sos@yahoo.gr; 3 Vascular Unit, First Department of Propedeutic Surgery, National and Kapodistrian University of Athens, 157 72 Athens, Greece; georgegalyfos@hotmail.com; 4 Clinical, Experimental Surgery & Translational Research, Biomedical Research Foundation Academy of Athens, 115 27 Athens, Greece; vekrellis@bioacademy.gr (K.V.); nkostom@bioacademy.gr (N.K.); 5 Independent Researcher, 157 72 Athens, Greece; p.katafygiotis@gmail.com; 6 Department of Vascular Surgery, “Attikon” University Hospital, School of Medicine, National and Kapodistrian University of Athens, 157 72 Athens, Greece; andreaslazaris@hotmail.com (A.L.); ggeroulakos@med.uoa.gr (G.G.); liapis@med.uoa.gr (C.L.); kakisis@med.uoa.gr (J.K.)

**Keywords:** cilostazol, aspirin, stroke, MCAO model, hippocampus, diaschisis

## Abstract

Objective: The contralateral hippocampus, a critical region for cognitive function, is often overlooked in everyday clinical practice and stroke research. This study aimed to evaluate the effect of specific antiplatelet agents on the hippocampus (ipsilateral and contralateral) during the hyperacute phase of stroke. Materials and Methods: Twelve-week-old rats were randomly assigned to four groups, each containing six rats: a cilostazol group, an aspirin group, an aspirin plus cilostazol group, and a control group. Each substance was administered for four weeks. Permanent brain ischemia was induced over 2 h using intraluminal middle cerebral artery occlusion. A neurologic examination was conducted, followed by euthanasia and histological examination of the CA1 hippocampal region. The hematoxylin and eosin stain was used to assess the total number of intact neuronal cell bodies and pyknotic nuclei, an indicator of early irreversible neuronal injury. Results: In the ipsilateral hippocampus, monotherapy with either aspirin or cilostazol significantly reduced pyknotic nuclei compared with the control group (*p* = 0.0016 and *p* = 0.0165, respectively). However, combination therapy showed no significant difference from the controls (*p* = 0.2375). In the contralateral hippocampus, cilostazol monotherapy demonstrated significantly reduced pyknotic nuclei (*p* = 0.0098), whereas aspirin monotherapy and combination therapy did not (*p* = 0.1009 and *p* = 0.9999, respectively). A cumulative analysis of both hemispheres revealed that monotherapy with aspirin or cilostazol markedly reduced injury markers (*p* = 0.0002 and *p* = 0.0001, respectively), whereas combined therapy revealed no significant benefit (*p* = 0.1984). A neurological assessment indicated that the most severe deficits were in the combination therapy group. Conclusions: To the best of our knowledge, this is the first study to compare acute histopathological changes in the affected and unaffected hippocampus after a stroke in a rat model. Dual antiplatelet therapy resulted in worse outcomes (histopathological and neurological) than monotherapy.

## 1. Introduction

Cerebrovascular accidents (CVAs) are debilitating events characterized by the sudden deterioration of blood flow to a brain region, resulting in morphological and pathophysiological disturbances that can lead to neurodegeneration and permanent neurological deficits. Two highly important mechanisms for recovering functions in the early phase after a stroke are the reperfusion of the ischemic penumbra and the resolution of diaschisis [1,2]. Although reperfusion has been predominantly investigated, the pharmacological resolution of diaschisis has not been thoroughly studied. The contralateral hemisphere—specifically, the contralateral hippocampus—is a region of the brain usually neglected in everyday clinical practice and stroke research. In the same vein, cilostazol, which possesses antiplatelet, vasodilatory, anti-inflammatory, and neuroprotective effects, could theoretically contribute to the non-invasive prevention of brain ischemia in both hemispheres during the hyperacute phase [3,4].

The hippocampus is a special brain structure mainly involved in memory and learning, and it is very sensitive to ischemia. Neuroplasticity is known to occur in this area, and cilostazol seems to help treat it [5]. In this experimental study, we evaluated the effectiveness of different therapeutic agents (cilostazol, aspirin, and combined therapy) on the hippocampus in the hyperacute phase after a stroke, both ipsilaterally and contralaterally.

## 2. Material and Methods

Twelve-week-old healthy male Sprague-Dawley rats were randomly assigned to four groups, each containing six rats (n = 6): a control group (normal saline), a cilostazol group (30 mg/kg/daily), an aspirin group (10 mg/kg/daily), and an aspirin plus cilostazol group (10 mg/kg/daily and 30 mg/kg/daily, respectively). Each substance was administered via gavage for four weeks. All animals were subjected to brain ischemia for 2 h using intraluminal middle cerebral artery occlusion. A neurologic examination was performed, and the animals were then sacrificed. The aforementioned dosages for aspirin and cilostazol were based on their pharmacokinetic properties, therapeutic efficacy, and safety profiles, as demonstrated in preclinical studies [3,6].

This methodology was employed to assess the effect of cilostazol and other pharmacological agents on hyperacute diaschisis in the hippocampus. After euthanasia, brain tissues were carefully removed from the skull and then fixated and prepared for a histological examination of the CA1 region of the hippocampus. Sections from both hemispheres were assessed for the total number of intact neuronal cell bodies (with normal cell membrane nuclei with even staining) and pyknotic nuclei (small, shrunken, and darkly stained), an early sign of irreversible neuronal injury [7].

### 2.1. Ethics Statement

The experimental protocol was approved by the “Scientific Committee for the Approval of Protocols Using Animals for Scientific Purposes” (Decision No. 602325/08-10-2019), established at the Laboratory for Experimental Surgery and Surgical Research “Biomedical Research Foundation Academy of Athens” of Athens Medical School, and by the Veterinary Directorate of the Attica Region. In compliance with local animal research protection regulations, this study was designed and carried out to reduce total animal sacrifice and minimize distress before, during, and after the operation. Any signs of complications or adverse reactions were addressed promptly, and additional veterinary care was provided if required. Detailed records and overall recovery progress were maintained for each animal.

### 2.2. Drug Delivery

All substances were diluted in normal saline and administered orally for four weeks prior to cerebral permanent ischemia (volume of approximately 1 mL). The concentration and duration of the administration of the substances were based on the pharmacokinetics of cilostazol. Although cilostazol requires approximately four weeks to achieve its optimal therapeutic effect, compared with only a few days for aspirin, both medications were initiated simultaneously and administered for the same duration. The aspirin and cilostazol dosages were based on their pharmacokinetic properties, therapeutic efficacy, and safety profiles, as demonstrated in preclinical studies. These dosages are commonly used in experimental research to reflect the therapeutic effects observed in human studies [3,6].

### 2.3. Surgical Procedure

Rats were fasted but given free access to water 12 h prior to surgery. Each animal was anesthetized with isoflurane via inhalation at a dose of 2.0–3.0 ml/L. Each rat was immobilized in the supine position on a surgical table, and the left common, internal, and external carotid arteries were exposed and carefully dissected, avoiding injury to soft tissues and nerves. Microsurgical clamps were placed on the common and internal carotid arteries near the bifurcation, and the ECA was partially incised near the bifurcation. A silicone rubber-coated monofilament suture (Doccol Corporation, Sharon, MA, USA) was advanced into the ECA lumen. The clamp of the ICA was removed, and a nylon suture was advanced from the ECA to the ICA lumen; the suture was advanced into the ICA until resistance was felt. At this point, the intraluminal suture blocked the origin of the middle cerebral artery. The occlusion time was recorded with a timer. The incision layers were closed, and the animals were resuscitated. Following the permanent MCAO procedure and recovery from anesthesia, the rats were monitored closely in a recovery area where they were observed for signs of pain, distress, or other postoperative complications. A neurological examination was conducted 120 min after the rats regained consciousness and mobility.

### 2.4. Neurological Examination

Following the complete recovery of the rats, we assessed motor deficits and neurological impairments using the Longa Score, a 5-point scoring system. Score 0 indicates no observable deficits; score 1 indicates failure to extend the contralateral forelimb fully (mild motor impairment); score 2 indicates circling behavior toward the contralateral side but normal posture at rest (moderate motor impairment); score 3 indicates spontaneous circling at rest (significant motor impairment); and score 4 indicates loss of consciousness or no spontaneous motor activity (severe neurological deficit). The authors received comprehensive training on the Longa Score assessment method prior to conducting the experiments to ensure competency in interpreting phenotypes.

### 2.5. Histological Examination

**Figure 1:**
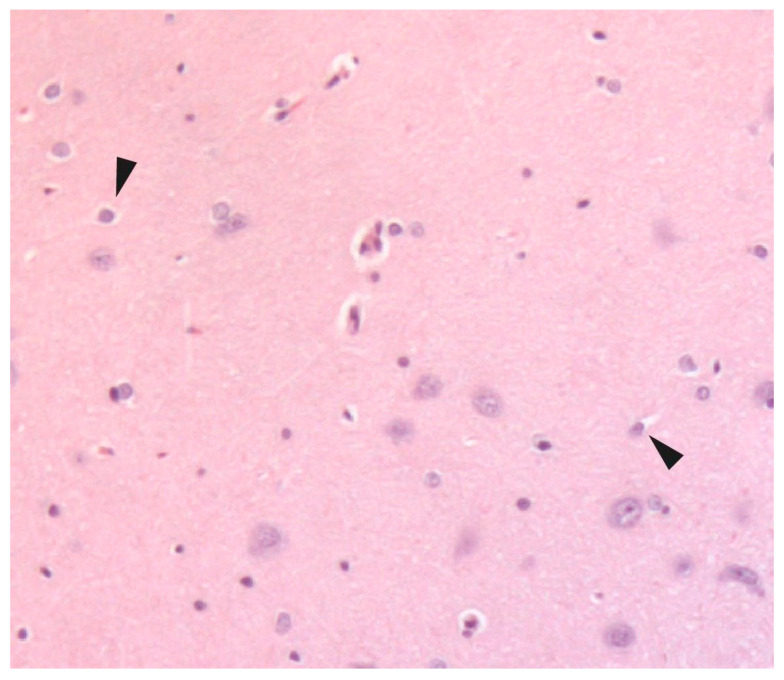
Representative histological appearance of rat brain hippocampus with hematoxylin and eosin (H&E). Tiled photomicrograph of a coronal section of a rat brain showing the hippocampal region. The arrowhead in the framed area shows ischemic neuronal damage (×20).

**Figure 2:**
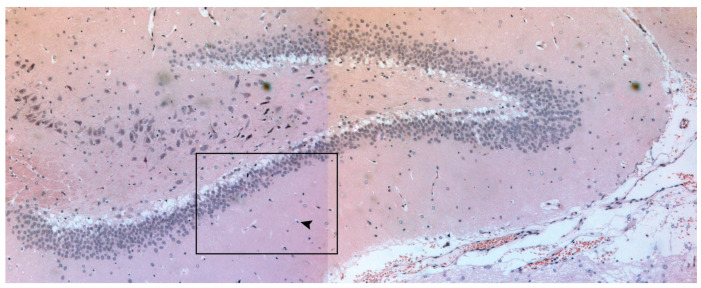
Light microscopy image ×40 of the CA1 hippocampal area depicting neuronal death of hippocampal pyramidal cells. The arrowheads identify neuronal cytoplasmic shrinkage accompanied by pyknotic nuclei. Within the black box (granule cell layer and the adjacent hilus) there are numerous pyknotic nuclei—characterized by their small, dark, and densely stained appearance.

After euthanasia, brain tissues were carefully removed from the skull and immediately fixed in 10% neutral buffered formalin (NBF) at room temperature for 24 h. After fixation, the samples were processed and embedded in paraffin. To assess necrotic neuronal nuclei in the rat hippocampus, 5 μm thick coronal-paraffin-embedded hippocampal sections were collected and stained with hematoxylin and eosin [8,9]. The sections were placed on the automated stage of a LEICA DMRA2 upright microscope, and the tile scan images (20× magnification) of the rat hippocampus were obtained using Stereo Investigator v10.0 (MBF Bioscience, Williston, VT, USA). The necrotic neuronal nuclei (small, shrunken, and darkly stained) were counted using the “cell counter” module of Fiji v2.0.0. These pyknotic nuclei are an early sign of irreversible neuronal injury [7]. The total necrotic nuclei (with normal cell membrane nuclei with even staining) per section were normalized to the analyzed section surface (graph: axis → total number of necrotic nuclei/surface area) (Figure 1 and Figure 2).

### 2.6. Statistical Analysis

Necrotic-to-total nuclei percentage ratios were calculated for data normalization. Non-parametric multiple comparisons (Kruskal–Wallis test) were employed to assess the mean rank difference and statistical significance of the control vs. aspirin, cilostazol, and combinatorial treatments. The correlation of dependent variables (necrotic-to-total nuclei % ratio) with treatment arms and neurological examination findings (mild, moderate, or severe) was assessed using multiple linear regression (sum of squares, 215.4; degrees of freedom, 6; mean squares, 35.91). The normality of residuals was assessed using the D’Agostino–Pearson omnibus (K2), passing the normality test. The resource equation approach was employed ad hoc to ensure sample size adequacy. Post hoc analysis was also conducted on the measured mean and standard deviation values per group to ensure statistical integrity.

## 3. Results

**Figure 3:**
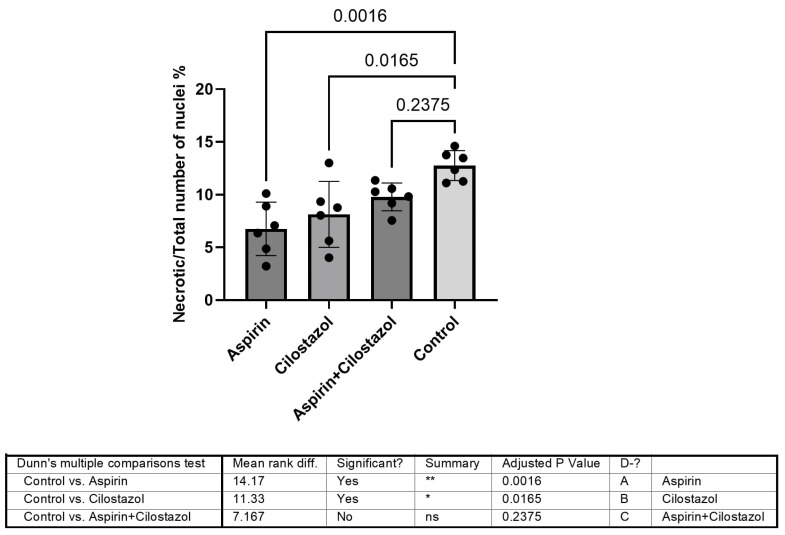
Data from the ischemic hippocampus revealed that the monotherapy groups (aspirin or cilostazol) differed significantly from the control group, whereas the dual antiplatelet group did not differ significantly from the control group. ns is not significant, * is very significant (*p* < 0.05), ** is highly significant (*p* < 0.01).

A comparison of the ipsilateral ischemic (left) hippocampus data revealed that the monotherapy group, either with aspirin or with cilostazol, differed significantly from the control group (*p* = 0.0016 and *p* = 0.0165, respectively). The combination of drugs (the cilostazol plus aspirin group) exhibited a higher ratio of pyknotic-to-total nuclei, which was not significantly different from the control group (*p* = 0.2375) (Figure 3).

**Figure 4:**
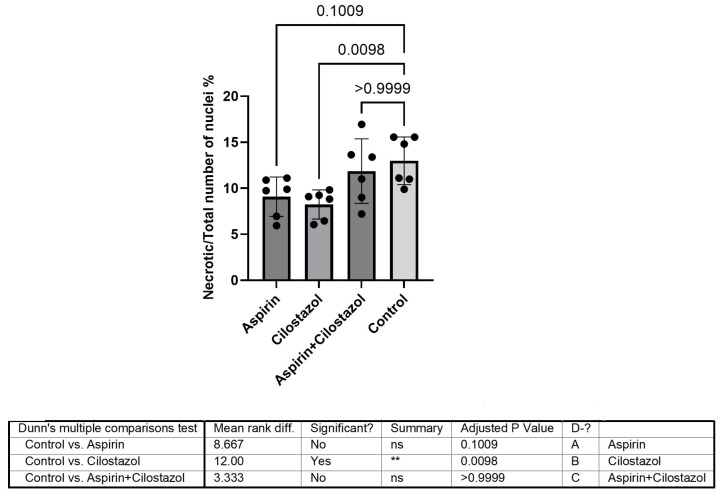
Data from the non-ischemic hippocampus revealed that the cilostazol group demonstrated significantly lower outcomes than the control group, whereas the aspirin and combined therapy (cilostazol–aspirin) groups did not differ significantly from the control group. ns is not significant, ** is very significant (*p* < 0.01).

The contralateral non-ischemic (right) hippocampus comparison of groups demonstrated that the ratio of pyknotic-to-total nuclei in the cilostazol group had significantly lower outcomes than the control group (*p* = 0.0098). The aspirin and combined therapy (cilostazol/aspirin) groups exhibited higher ratios of pyknotic-to-total nuclei, which was not significantly different from the control group (*p* = 0.1009 and *p* = 0.9999, respectively) (Figure 4).

**Figure 5:**
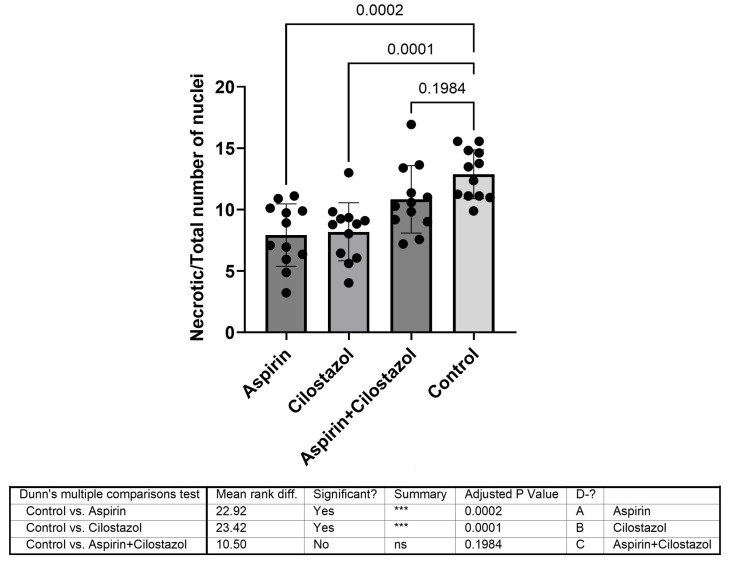
Cumulative data from both hemispheres. Monotherapy with either aspirin or cilostazol showed lower levels than the control group, whereas the dual antiplatelet therapy group did not demonstrate a significantly different ratio from that of the control group. ns is not significant, *** is highly significant (*p* < 0.001).

Furthermore, the cumulative data from both hemispheres were compared between the groups. Monotherapy with either aspirin or cilostazol showed markedly lower levels than the control group (*p* = 0.0002 and *p* = 0.0001, respectively), whereas the dual antiplatelet therapy revealed no significant ratio compared with the control group (*p* = 0.1984) (Figure 5).

The correlation between the histopathologic findings and the neurological assessment revealed that the dual antiplatelet therapy group experienced the most severe neurological deficits (Table 1).

## 4. Discussion

This study addresses the knowledge gap regarding the effects of cilostazol, alone or in combination with aspirin, during the very early phase after a CVA. The pleiotropic characteristics of cilostazol led us to investigate a combination of therapies in the very acute phase in the hippocampus, an organ correlated with cognitive function. According to our results, cilostazol fails to express neuroprotective results in the very acute phase after a stroke when combined with aspirin. This should be further investigated to define the proper treatment strategy for patients suffering from stroke while on this specific regimen or in patients with significant carotid artery occlusion.

In this study, we caused permanent rather than transient ischemia for two reasons. First, the transient ischemia model is better for causing reperfusion in the brain, which we wanted to avoid [10]. Our focus was on pharmacological contributions (cilostazol and aspirin) rather than reperfusion of the penumbra with either treatment. Secondly, reperfusion can induce oxidative stress and inflammation, which can lead to more severe blood-brain barrier (BBB) incompetence and, therefore, different pathology findings [11,12]. Moreover, we chose to include cilostazol in the study because it has not been comprehensively studied regarding CVAs and because it is believed to have neuroprotective effects [3,13,14,15]. However, we observed that monotherapy with either aspirin or cilostazol comes with better outcomes in the ipsilateral hemispheres and the overall data from both hemispheres than a combination of treatments. Specifically, cilostazol failed to preserve its neuroprotective effects in the very early phase after a stroke when delivered with aspirin. This effect could arise from several mechanisms, such as a conflict in pharmacodynamic interaction (either in their antiplatelet or anti-inflammatory effects) or due to the timing of co-administration (e.g., cilostazol may be beneficial after a stroke but not before or during the hyperacute phase).

The pathophysiologic processes after CVA are categorized into different timeframes: the very early or hyperacute phase (minutes to 6 h), the acute phase (up to the first week), the subacute phase (1 week to several months), and the chronic phase (months to years) [16]. We focused on the hyperacute phase, as it is correlated with cascades like excitotoxicity, oxidative stress, and disruption of the BBB. Although this classification is based on clinical timelines and studies, similar windows have been applied in experimental research on rodents. We selected the specific timeframe to assess the immediate effect of therapeutic intervention because it is considered the most critical for immediate intervention. Yu Ri Ki et al. found that cilostazol was associated with the inhibition of neurodegeneration and an anti-depressant effect after a stroke [4]. It would be significant if a preventive pharmacological substance could ameliorate clinical and pathological findings in the hyperacute phase. Our results revealed that aspirin and cilostazol have similar effects on the ipsilateral hippocampus and that combinational treatments do not offer additional benefits, at least in the very early phase.

Cilostazol, a phosphodiesterase III inhibitor, increases intracellular cAMP levels [17] leading to the activation of cAMP-response element-binding protein (CREB), which regulates genes involved in neuronal survival and plasticity. It also inhibits JNK3/caspase-3 by enhancing Akt1, contributing to improved neural plasticity and cognitive function [13,14]. These effects suggest that cilostazol exerts both vascular and neuroprotective actions. In contrast, aspirin’s primary mechanism involves the inhibition of cyclooxygenase, thereby blocking thromboxane A2 synthesis and preventing collagen-induced platelet aggregation [18]. However, aspirin does not modulate neuronal signaling pathways or anti-apoptotic cascades. Cilostazol inhibits a broader ranger of aggregation stimuli (primary and secondary platelet aggregation caused by aggregating agents such as adenosine diphosphate (ADP)) than aspirin. Combined therapy with both drugs is thought to enhance antiplatelet efficacy, which is not correlated with an increased risk of bleeding or other complications, as supported by several studies [19,20]. However, in our study, the dual antiplatelet group did not demonstrate any benefit in the hyperacute phase and, in fact, may be correlated with unfavorable outcomes compared with monotherapy. One explanation could be excessive platelet inhibition, which may have led to hemorrhagic transformation. To confirm this assumption, one should look for real signs of hemorrhage, confirmed with histopathologic or radiology findings in a subsequent stroke phase. None of these were directly assessed in our study due to methodological limitations. To date, only two observational studies have revealed this possible adverse event in patients with stroke [21,22].

Often observed following brain ischemia, diaschisis is a phenomenon in which neurophysiological disturbances occur in regions that are distant from the initial injury [23]. It is mainly documented during the subacute and chronic phases after a stroke and is correlated with cognitive impairment [24,25]. Little is known about the very early phase after a stroke, and this is what we aimed to shed light on. According to our findings, cilostazol demonstrated better outcomes than aspirin; however, it is unclear whether this result is stochastic and whether it persists in the following stroke phases. Therefore, cilostazol may offer a compensatory mechanism for the contralateral hippocampus, which is somehow damaged when dual antiplatelet treatments with aspirin are administered. Widespread pyknotic nuclei could be explained by several phenomena related to interhemispheric injuries, such as post-ischemic depolarization, transcallosal effects, overexcitation, transsynaptic effects, bilateral blood-brain barrier opening, and edema [10].

The hippocampus is a small brain structure involved in learning and memory functions composed of subregions CA1, CA2, and CA3. A growing number of studies are investigating the effect of CVAs on different areas of the brain, including the CA1 region of the hippocampus because of its vulnerability to ischemic damage. DISCOVERY, for example, is an ongoing, prospective, multicenter, observational study investigating the mechanisms of susceptibility to post-stroke cognitive impairment and dementia. A potential treatment target may be the preservation of the hippocampal vascular structure and circulatory dynamics, which still need to be thoroughly investigated. In this context, Afsaneh Asgari Taei et al. reported that human embryonic stem cell-derived mesenchymal stem cells could promote hippocampal neurogenesis and angiogenesis concomitant by inhibiting inflammation and apoptosis in ischemic brains [26]. Other unconventional strategies have also been studied, such as in one study suggesting that Pluchea lanceolata, a herb used in traditional medicine, preserves cognitive function and pyramidal neurons in the CA1 and CA3 regions, leaving the dendritic architecture intact after induced ischemia [27]. In our study, we examined the CA1 region to maintain a focused analysis.

A critical pathophysiology mechanism after acute stroke is the disruption of the BBB. The combined use of cilostazol and aspirin may synergistically exacerbate BBB disruption, potentially contributing to increased apoptosis compared with monotherapy. Cilostazol increases intracellular cAMP and activates protein kinase A, which typically reduces intracellular Ca^2+^, causing vasodilatory effects [17]. Aspirin irreversibly inhibits the enzyme cyclooxygenase-1 and thus suppresses thromboxane A2 synthesis. By inhibiting thromboxane production, aspirin also reduces vasoconstriction [28,29]. The combined vasodilatory effect might increase hydrostatic pressure in the cerebral vessels, potentially leading to vasogenic edema. This edema could induce excessive cell stress, promoting early apoptosis. However, our findings should be interpreted with caution since our study did not investigate the BBB or the presence of cerebral edema.

### Limitations of This Study

Our study has several limitations. Firstly, histopathologic findings must be considered in conjunction with other diagnostic tests (e.g., magnetic resonance imaging) or functional and behavioral tests (such as the beam walk or Morris water maze tests) to measure the extent of brain tissue damage. Secondly, the consequences of lacunar or other stroke types differ from those observed in the monofilament MCAO model; therefore, we cannot conclude whether dual antiplatelet therapy with cilostazol is beneficial in other types of strokes. This type of model is not suitable for investigating secondary prevention but rather primary prevention and acute treatment. Another limitation of the current study is the absence of molecular and cellular marker validation (such as GFAP, caspase-3, or NeuN) and infarct volume measurements, both of which would have provided a more detailed understanding of the extent of ischemic damage. However, to determine infarct volume, one needs to reliably distinguish between infarct and adjacent tissue, which is typically only possible after 24 h. In the permanent MCAO model, the 24 h survival rate is low, limiting this assessment. As an alternative, we used the necrotic-to-total nuclei percentage ratio to indirectly determine the severity of stroke despite the absence of an infarct volume. Lastly, we hypothesize that the reduced improvement observed with dual antiplatelet therapy is related to increased blood-brain barrier (BBB) disruption, potentially leading to brain edema. However, we did not directly assess BBB integrity or measure cerebral edema, and thus, this interpretation remains speculative. Future studies incorporating quantitative assessments of BBB permeability and brain water content (e.g., via Evans Blue extravasation or brain water content measurement) would be valuable in testing this hypothesis.

## 5. Conclusions

To the best of our knowledge, this is the first report comparing very acute changes in the affected and unaffected hippocampus after CVA in a permanent focal ischemic rat model, where rats were treated with cilostazol, aspirin, or a combination of both. The combinational treatment exhibited worse outcomes than monotherapy in terms of early apoptosis (pyknotic nuclei) and neurological outcomes. There may be conflicting mechanisms of action at the cellular level of those two substances in the CVA setting that have yet to be elucidated. Researchers studying acute brain ischemia and physicians managing patients with multi-vascular disease should anticipate further studies to confirm or reject these findings.

## Figures and Tables

**Table 1:** *p*-value summary: ns represent not significant, *** is highly significance (*p* < 0.001), **** is highly significance (*p* < 0.0001).

Parameter Estimates	Variable	Estimate	Standard Error	95% CI (Asymptotic)	|t|	*p*-Value	*p*-Value Summary
β0	Intercept	11.77	1.128	9.494 to 14.05	10.44	<0.0001	****
β1	Neurologic Examination [4]	0.5444	0.9294	−1.333 to 2.421	0.5858	0.5612	ns
β2	Neurologic Examination [3]	1.659	1.079	−0.5198 to 3.837	1.538	0.1318	ns
β3	Neurologic Examination [1]	−0.4031	1.401	−3.233 to 2.427	0.2877	0.7750	ns
β4	Team [1]	−4.306	1.095	−6.517 to −2.094	3.932	0.0003	***
β5	Team [2]	−4.157	1.080	−6.337 to −1.976	3.850	0.0004	***
β6	Team [3]	−1.054	1.166	−3.408 to 1.301	0.9037	0.3714	ns

## Data Availability

You can contact the author to ask for the original data based on reasonable request.
